# Myoclonus ataxia and refractory coeliac disease

**DOI:** 10.1186/2053-8871-1-11

**Published:** 2014-09-01

**Authors:** Ptolemaios G Sarrigiannis, Nigel Hoggard, Daniel Aeschlimann, David S Sanders, Richard A Grünewald, Zoe C Unwin, Marios Hadjivassiliou

**Affiliations:** Departments of Neurophysiology, Royal Hallamshire Hospital, Sheffield, S10 2JF UK; Neuroradiology, Royal Hallamshire Hospital, Sheffield, S10 2JF UK; Neurology, Royal Hallamshire Hospital, Sheffield, S10 2JF UK; Gastroenterology, Royal Hallamshire Hospital, Sheffield, S10 2JF UK; College of Biomedical and Life Sciences, Cardiff University, Cardiff, CF14 4XY UK

**Keywords:** Myoclonus, Ataxia, Tremor, Epilepsy, EEG, Refractory, Coeliac

## Abstract

**Background:**

Cortical myoclonus with ataxia has only rarely been reported in association with Coeliac Disease (CD). Such reports also suggested that it is unresponsive to gluten-free diet. We present detailed electro-clinical characteristics of a new syndrome of progressive cortical hyperexcitability with ataxia and refractory CD. At our gluten/neurology clinic we have assessed and regularly follow up over 600 patients with neurological manifestations due to gluten sensitivity. We have identified 9 patients with this syndrome.

**Results:**

All 9 patients (6 male, 3 female) experienced asymmetrical irregular myoclonus involving one or more limbs and sometimes face. This was often stimulus sensitive and became more widespread over time. Three patients had a history of Jacksonian march and five had at least one secondarily generalised seizure. Electrophysiology showed evidence of cortical myoclonus. Three had a phenotype of epilepsia partialis continua at onset. There was clinical, imaging and/or pathological evidence of cerebellar involvement in all cases. All patients adhered to a strict gluten-free diet with elimination of gluten-related antibodies in most. However, there was still evidence of enteropathy in all, suggestive of refractory celiac disease. Two died from enteropathy-associated lymphoma and one from status epilepticus. Five patients were treated with mycophenolate and one in addition with rituximab and IV immunoglobulins. Their ataxia and enteropathy improved but myoclonus remained the most disabling feature of their illness.

**Conclusions:**

This syndrome may well be the commonest neurological manifestation of refractory CD. The clinical involvement, apart from ataxia, covers the whole clinical spectrum of cortical myoclonus.

**Electronic supplementary material:**

The online version of this article (doi:10.1186/2053-8871-1-11) contains supplementary material, which is available to authorized users.

## Background

The term gluten-related disorders (GRD) encompasses a spectrum of intestinal and extra-intestinal manifestations that are immune-mediated and triggered by gluten ingestion [1]. Neurological manifestations are increasingly recognised with gluten ataxia (GA) being the best characterized entity [2]. Unlike GA, ataxia with myoclonus and celiac disease (CD) is a rare entity first reported in 1966 [3].

A subsequent case report [4], described a patient with CD, ataxia and tremor of the eyelids, chin and palate. The pathology showed cerebellar cortical atrophy and cell loss in dentate and olivary nuclei. Another report [5] described a patient with established CD, cerebellar ataxia and widespread myoclonus with neuropathological evidence of Purkinje cell loss.

In 1986 Lu and colleagues published two cases with action myoclonus, ataxia and CD who in addition had epilepsy [6]. The authors provided electrophysiological evidence for the cortical origin of the myoclonus. Similar findings of action, stimulus sensitive, cortical myoclonus were subsequently reported in another patient [7]. This patient had cortical reflex and action myoclonus resembling epilepsia partialis continua, with constant arrhythmic myoclonic activity in the right hypothenar muscles. Electrophysiology confirmed the cortical origin of the myoclonus.

The largest case series was published in 1995 [8] and reported 4 patients with myoclonus and ataxia with electrophysiological evidence of stimulus sensitive myoclonus of cortical origin. Pathology showed atrophy of the cerebellar hemispheres with Purkinje cell loss. CD was diagnosed in all four, preceding the onset of the neurological manifestations by years. Further individual case reports [9, 10] appeared at a later stage but no large series.

Such patients unlike those with gluten ataxia appear to be poorly responsive to gluten-free diet and follow a progressive course. The type of enteropathy (i.e. refractory versus gluten responsive), the serological characterization and any response to immunosupression has never been investigated or reported.

At the gluten/neurology clinic of our institution (Royal Hallamshire Hospital, Sheffield, UK) we have assessed and regularly follow up over 600 patients with neurological manifestations of GRD. We have identified 9 patients with this syndrome confirming that this is rare entity but possibly under-diagnosed. In this report we outline the electro-clinical and imaging findings (including MR spectroscopy), bowel histology and serological findings as well as our experience with immunosuppressive and symptomatic treatment.

Patients underwent EEG and polygraphic surface EMG recordings, SEPs, assessment for C-reflexes and jerk-locked back averaging (JLBA). The latter was performed based on previously published studies [11, 12] and the SEPs in line with recent reccomandations [13]. A summary of the clinical features of the myoclonus and the neurophysiological findings are found in Table 1. Further clinical, serological and histopathological (duodenal biopsy) data is summarised in Table 2.

**Table 1:** **Clinical and electrophysiological findings in 9 patients with myoclonic ataxia and Coeliac disease**

	Age*		
	Sex	Clinical features of myoclonus	Electrophysiology
**1**	50/M	EPC right face and tongue (5-6 Hz). Episodes of Jacksonian march, spreading from face into platysma and right shoulder, occasionally leading to secondary generalised tonic-clonic seizures. EPC attenuated during sleep.	Normal EEG, SEPs, no LLRs. JLBA, revealed that right facial/tongue twitching was EPC (Figure 2). Normal blink reflex studies and no signs of denervation on affected facial and tongue muscles. Ten years from onset of neurological symptoms, axonal PN on EMG & NCS.
**2**	60/F	Continuous myoclonic tremor at around 5 Hz of the right arm. Occasional myoclonus of the right leg. The left arm developed asynchronous (5-7 Hz) myoclonic tremor at a later stage. A single secondary generalised seizure.	Standard EEG unremarkable, SEPs (only left hand) within normal limits. JLBA, cortical myoclonic tremor of right UL (Additional file 1: Figure S1). JLBA at a later stage revealed cortical myoclonic tremor on the left UL. Normal NCS.
**3**	63/M	Continuous myoclonic jerks/action myoclonus of the right UL (5 Hz). Two years later, deterioration with facial twitching and prominent action myoclonus (R leg)	Mild excess of widespread theta and occasionally delta range activity, maximal in the temporal and centrotemporal regions. JLBA, continuous cortical myoclonus (right hand). ‘Giant’ SEPs, right leg, but no LLR (Additional file 2: Figure S2). Normal NCS.
**4**	53/M	Very frequent irregular spontaneous, action and reflex myoclonic tremor of left UL. Later on, episodes of Jacksonian march and secondary generalisation.	JLBA, spontaneous and action induced cortical myoclonus. Giant SEPs from ULs with LLRs, better formed on the right, clinically less affected side (Figure 4).
**5**	76/M	Irregular myoclonic action tremor of both ULs (L > R). Spontaneous and reflex myoclonic tremor of the intrinsic hand muscles (L > R at ~5-6 Hz).	JLBA, cortical origin of the very frequent spontaneous myoclonus of the right intrinsic hand muscles and forearm. Low amplitude LLR from left UL (Additional file 2: Figure S2). NCS & EMG, axonal sensorimotor PN.
**6**	46/F	Irregular spontaneous but mainly action and reflex myoclonic tremor of both UL and LL.	‘Giant’ cortical SEPs and LLRs from both median and the right tibial nerves. JLBA, cortical origin of spontaneous and action induced myoclonus (Figure 4).
**7**	61/M	Myoclonic status epilepticus with twitching of L UL and LL.	Standard EEG, PLEDs in the right posterior quadrant with irregular not time-locked asynchronous myoclonic jerks of the left upper and lower limb. JLBA, cortical generator for the myoclonus (Figure 1).
**8**	52/F	Spontaneous, action and reflex myoclonus of ULs (L > R at ~10 Hz).	JLBA, spontaneous and action induced cortical myoclonus. ‘Giant’ SEPs from both ULs (L > R) and LLRs (Figure 4).
**9**	74/M	Mainly action and reflex myoclonus of LLs (L > R at ~ 4 Hz)	JLBA, mainly action and reflex cortical myoclonus. ‘Giant’ SEPs only from left LL plus LLRs (Additional file 3: Figure S3).

CD = coeliac disease, EEG = electroencephalogram, EPC = epilepsia partialis continua, EMG = electromyography, JLBA = jerk-locked back averaging, LLR = long loop reflexes, NCS = nerve conduction studies, PLED = periodic lateralised epileptiform discharge, PN = peripheral neuropathy, SEP = somatosensory evoked potentials, UL = upper limb, LL = lower limb.

*Age at onset of neurological manifestations.

**Table 2:** **Summary of serological and histopathological characteristics of the 9 patients**

Case	Age/sex	Gluten related antibodies baseline				Gluten related antibodies on strict gluten-free diet				Duodenal biopsy baseline	Duodenal biopsy on diet (duration on diet in years)
		EMA	TG2	AGA	TG6	EMA	TG2	AGA	TG6		
**1**	50/M	n/a	n/a	n/a	n/a	-ve	-ve	-ve	-ve	Enteropathy	Enteropathy (10), EAL
**2**	60/F	+ve	n/a	+ve IgG	pos	-ve	-ve	-ve	-ve	Enteropathy	Enteropathy (5), type 1
**3**	63/M	+ve	+ve	+ve IgG, IgA	pos	-ve	-ve	-ve	-ve	Enteropathy	Enteropathy (3), type 1
**4**	53/M	n/a	n/a	n/a	n/a	-ve	-ve	-ve	-ve	Enteropathy	Enteropathy (10), type 2
**5**	76/M	+ve	+ve	+ve IgA	pos	-ve	-ve	-ve	-ve	Enteropathy	Enteropathy (2), type 1
**6**	46/F	+ve	+ve	n/a	n/a	+ve	-ve	+ve IgG	-ve	Enteropathy	Enteropathy (2), type 1
**7**	61/M	+ve	+ve	+ve IgG, IgA	n/a	-ve	+ve	+ve IgG	-ve	Enteropathy	Enteropathy (1), type 1
**8**	52/F	-ve	+ve	+ve IgG, IgA	pos	-ve	+ve	+ve IgG,IgA	-ve	Enteropathy	Enteropathy (1), type 1
**9**	74/M	+ve	+ve	n/a	n/a	-ve	-ve	+IgA	-ve	Enteropathy	Enteropathy (1.5), type 1

EMA = endomysium antibodies.TG2 = transglutaminase antibodies type 2. AGA = antigliadin antibodies. TG6 = transglutaminase antibodies type 6. EAL = enteropathy associated lymphoma. Enteropathy = triad of villous atrophy, crypt hyperplasia and increased intraepithelial lymphocytes. Type 1 enteropathy = refractory enteropathy. Type 2 enteropathy = refractory enteropathy with abnormal intraepithelial T cells.

## Results

### Clinical characteristics

**Figure 1:**
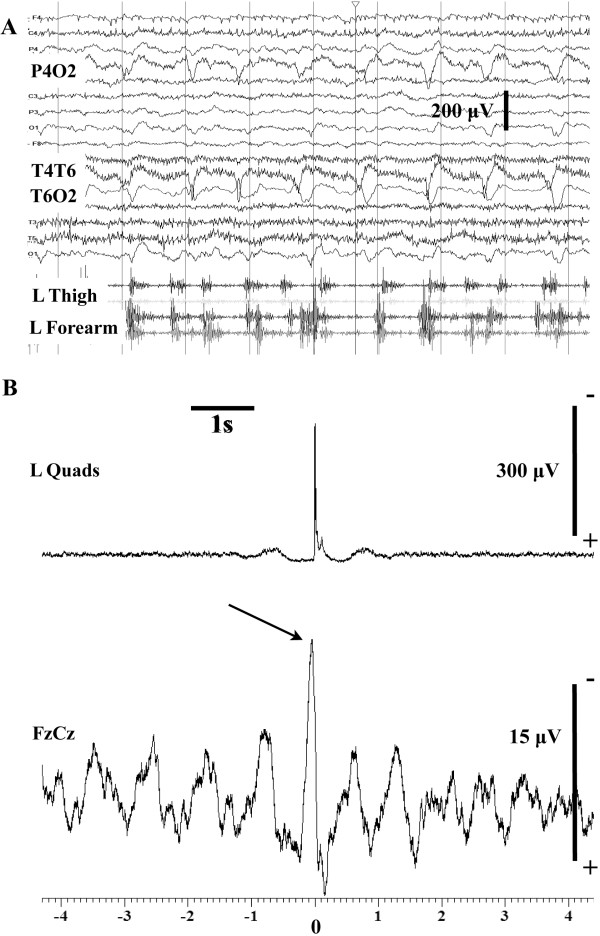
**Myoclonic status epilepticus (case 7). (A)** Routine EEG while the patient was obtunded and had frequent irregular left upper and lower limb myoclonic twitches. The surface EMG electrodes in the left thigh were recording from the quads (upper trace) and the biceps femoris. In the forearm, surface electrodes were recording from the extensor digitorum communis (upper trace) and the finger flexors. EEG tracing shows, about 1 Hz, periodic lateralized epileptiform discharges in the right posterior quadrant. Note that these are not time matched to the myoclonic jerks from the left arm and leg. **(B)** Jerk-locked back averaging from the left quads (80 sweeps). There is a time locked negative sharp wave preceding the onset of the averaged, rectified EMG data by about 25 ms. These recordings were performed in the ICU at a sampling rate of 500 Hz.

There were 9 patients (6 men, 3 women). Unlike previous case reports in all but 2 of our patients the neurological dysfunction was the initial presentation that lead to the diagnosis of CD. The mean age at onset of the neurological symptoms was 59 years (range 46–76). Unlike myoclonic ataxia (e.g. in the context of opsoclonus myoclonus ataxia syndrome) the myoclonic tremor in these patients was initially focal (face, tongue one arm and/or one leg) but then spread to affect other parts of the body. Epilepsy was a feature in 5 of the patients, 3 of which gave a history of Jacksonian march before progression to generalised seizures. However, only 3 of the patients had more than one seizure, one of which was the patient who presented with status epilepticus (Figure 1). Seizures were thus not a prominent feature and responded well to medication. All patients had a mild degree of limb and more prominent gait ataxia. This is in contrast to patients with GA where cerebellar ataxia is a very prominent and the presenting feature. Two patients developed axonal sensorimotor neuropathy. Gastrointestinal symptoms (including diarrhoea, weight loss, abdominal pain) were prominent in only 2 patients, in whom the diagnosis of CD was made prior to their neurological presentation.

#### Imaging findings

All patients underwent MRI scans including MR spectroscopy (7 patients) of the cerebellum (vermis and hemispheres). Mild cerebellar atrophy was noted in 8 patients. Abnormal spectroscopy of the cerebellum (reduced NAA/Cr ratio) was seen in all 7 patients who underwent spectroscopy (these included one patient with structurally normal cerebellum). In two patients, following the introduction of immunosuppression, repeat spectroscopy improved (increased NAA/Cr). Two patients had extensive white matter changes in a distribution suggestive of ischemia. The patient who presented with status epilepticus following the diagnosis of CD had high signal within the limbic system bilaterally the aetiology of which was thought to be the status epilepticus. PM examination in this patient showed loss of Purkinje cells in the cerebellum.

### Serological and histological characteristics

All apart from two patients had baseline serological testing, 7 of which showed positivity for endomysium and/or transglutaminase antibodies (>300 U/ml, normal range 0–15 U/ml) and 5 positive antigliadin antibodies. One patient had the biopsy without serological testing. Four baseline samples were available for testing for TG6 antibodies. All 4 were positive. All patients underwent gastroscopy and duodenal biopsy, 7 of which on more than one occasion. The baseline histology confirmed the presence of enteropathy with the triad of villous atrophy, crypt hyperplasia and increased intraepithelial lymphocytes in all 9 patients. Eight patients went on a strict gluten free diet with the mean duration of adherence to the diet being 3.7 years (range 1–10). One patient has only just been diagnosed and started the diet. Despite almost complete elimination of all antibodies repeat biopsies showed persistent enteropathy in 9 patients (7 refractory type 1 CD, 2 type 2 refractory CD). None of the above patients had any serological evidence of voltage gated potassium, calcium, NMDA or paraneoplastic antibodies. Full autoimmune profile was also normal.

### Effect of treatment and final outcome

All but one patient received treatment for their epilepsy and myoclonus. Medications used included clonazepam (up to 3 mg/day), sodium valproate (up to 2 g/day), lamotrigine (up to 800 mg/day), phenytoin (up to 400 mg/day), carbamazepine (up to 2 g/day), piracetam (up to 24 g/day), phenobarbitone (up to 180 mg/day), levetiracetam (up to 3 g/day), zonisamide (up to 400 mg/day) and more recently perampanel (up to 4 mg). The epilepsy was successfully controlled in all patients (free of secondarily generalised seizures) using just one of the above medications. The myoclonus however remained disabling despite maximum doses of individual drugs and the use of polytherapy. Immunosupression was used in 7 patients. This took the form of prednisolone and mycophenolate (7 patients). One patient who continued to progress on mycophenolate received in addition rituximab and intravenous immunoglobulins. In 2 patients the introduction of mycophenolate resulted in improvement of the ataxia both clinically and on MR spectroscopy of the cerebellum. Two other patients have only just started mycophenolate. The single patient who has tried several immunosuppressive treatments without any response remains extremely disabled and wheelchair bound due to the myoclonus although her most recent biopsy shows normalisation of the mucosa. The use of mycophenolate resulted in normalisation of the bowel mucosa in another patient. Two patients (both on mycophenolate) died, one as a result of biopsy proven metastatic enteropathy-associated lymphoma, the second with suspected enteropathy-associated lymphoma. The patient who presented with status epilepticus died of pneumonia. He had only received steroids. Recently 2 patients started perampanel with some significant improvement of their myoclonus.

#### Illustrative case history

This fifty-year-old man presented following a leg fracture after a trivial injury. Bone density scan demonstrated severe osteoporosis with low calcium and vitamin D. Two months later he noticed a persistent tremor affecting the right side of his mouth and lips including the tongue. He reported sudden onset of jaw locking associated with tongue biting and loss of consciousness. Past medical history included B12 deficiency.

**Figure 2:**
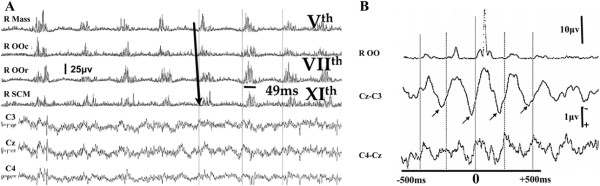
**Epilepsia partialis continua (case 1). (A)** Polygraphic EEG and EMG recordings. There is continuous ≈ 5 Hz synchronous rostral and caudal activation of brainstem innervated muscles. There is a fast rostrocaudal recruitment order, spreading from the upper pons into the bulbar region. The duration of the EMG discharges is below 50 ms. There are no EEG abnormalities in the central electrodes in the raw EEG recordings. **(B)** JLBA from the right OOr (2,100 sweeps) reveals rhythmical cortical correlates in the contralateral central region. They have a positive–negative morphology – the positive peak preceding the onset of EMG activity by ≈ 15 ms. Mass = Masseter, OOc = orbicularis oculi, OOr = orbicularis oris, SCM = sternocleidomastoid.

On examination he had right sided facial twitching (lower half of the face) involving lips and tongue (Figure 2). This interfered with his speech. He had mild gait ataxia.

He underwent gastroscopy and duodenal biopsy showing villous atrophy, crypt hyperplasia and increased intraepithelial lymphocytes in keeping with CD. He was started on a gluten-free diet and phenobarbitone.

A repeat duodenal biopsy performed 6 months later showed some improvement but persistent increase in the intraepithelial lymphocytes. In addition to phenobarbitone he was treated sequentially with sodium valproate, carbamazepine, clonazepam, levetiracetam, phenytoin, piracetam and botulinum toxin injections. The most beneficial interventions were the levetiracetam and the botulinum toxin injections though the facial twitching was never completely suppressed. His ataxia completely resolved a year after the commencement of a gluten-free diet.

He remained stable for the next 10 years apart from the twitching. He then complained of diarrhoea, weight loss, poor balance and numbness in his feet. He denied ingestion of gluten. He continued to have facial tremor with superimposed episodes of more severe shaking and jaw locking occurring with a frequency of 4 attacks per month. Neurological examination revealed lower limb and gait ataxia with absent ankle reflexes. He had reduction of sensation in his feet.

Serological testing for CD was negative. Repeat duodenal biopsy showed villous atrophy with crypt hyperplasia and increased intraepithelial lymphocytes. PAS staining was negative for Whipple’s disease. MR imaging showed cerebellar atrophy and low N-acetylaspartate to creatine ratio of the vermis and cerebellar hemispheres. CSF examination was normal including PCR for Whipple’s disease. Whole body PET scan was normal. A diagnosis of refractory CD type 1 was made. He was started on mycophenolate.

**Figure 3:**
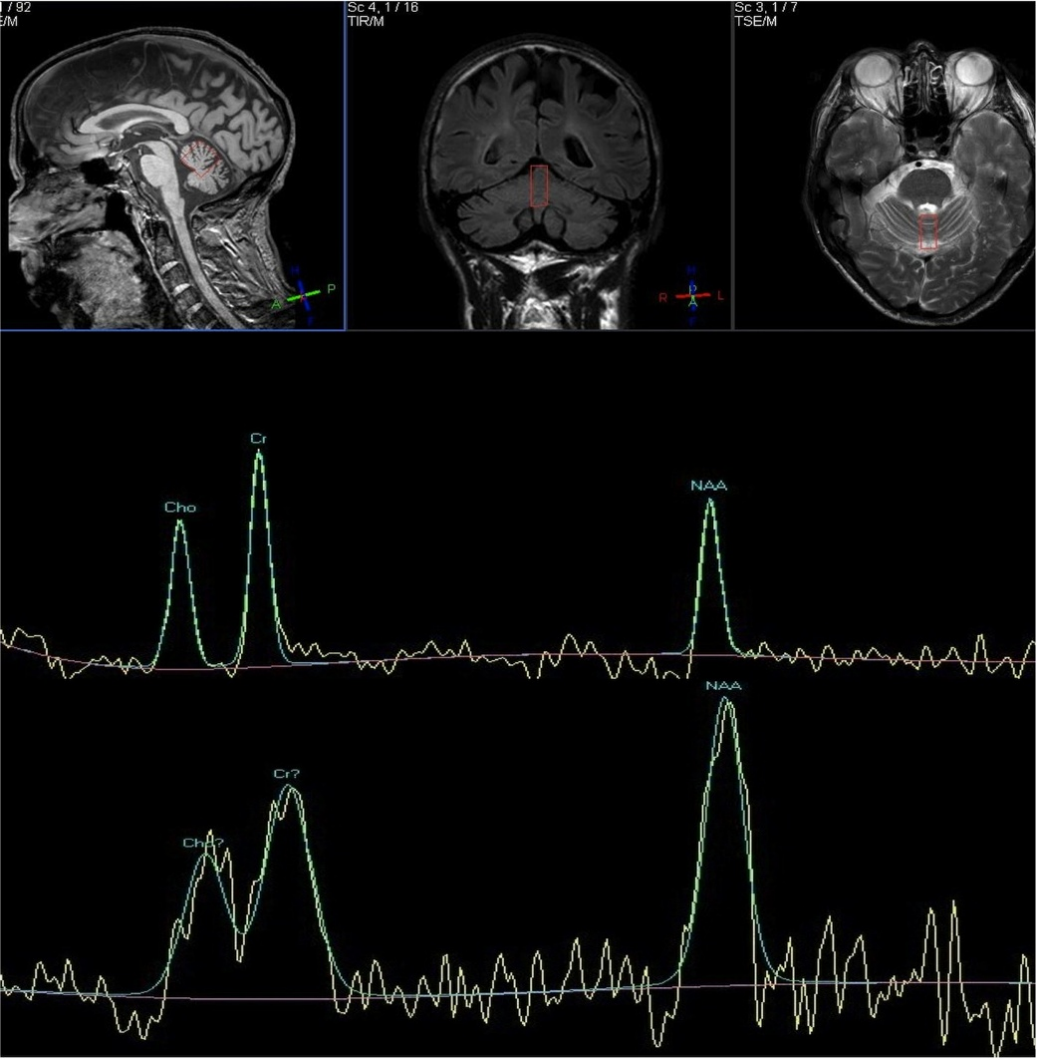
**MR spectroscopy (case1).** MR spectroscopy of the vermis before (upper trace) and 10 months after (lower trace) the introduction of mycophenolate. Patient already on gluten free diet for several years but with refractory coeliac disease (type 2). The NAA peak significantly increased as did the NAA/Cr (from 0.68 to 0.96). This was associated with clinical improvement of the ataxia.

Reassessment 10 months later showed significant improvement of the ataxia. This was associated with significant improvement of MR spectroscopy of the vermis (Figure 3). Antibody testing remained negative. The facial tremor persisted, and in addition he developed a tremor of the right arm. Nine months later he was admitted because of general malaise and weight loss. He had intermittent pyrexia with no source of infection. Due to pancytopenia the mycophenolate was stopped. He was treated empirically with antibiotics without any benefit. He underwent extensive investigations including brain MRI, gastroscopy and duodenal biopsy. There was no evidence of enteropathy. MRI showed cerebellar atrophy. PET scan showed multiple bony lesions suggestive of metastatic carcinoma. Bone biopsy showed T-cell lymphoma in keeping with enteropathy-associated lymphoma. He was too unwell to undergo chemotherapy and passed away a month after admission.

## Discussion

This report represents the largest series of patients with this unusual phenotype of “hyperexcitable” brain with cortical myoclonus, ataxia with refractory CD. Contrary to previous reports, in 7 of our 9 patients CD was diagnosed on the basis of their neurological presentation. We have demonstrated unequivocal electrophysiological evidence of the cortical origin for the myoclonus. In addition we presented, for the first time, evidence of refractory CD in all of our patients, with biopsy proven metastatic enteropathy-associated lymphoma in one and suspected enteropathy-associated lymphoma in another.

Refractory CD is a rare but well recognised entity that refers to those patients with CD who no longer respond to a strict gluten-free diet resulting in persistent symptoms of malabsorption and evidence of on-going enteropathy on repeat biopsies. It accounts for 10% of all patients with CD. Refractory CD is divided into 2 types. Refractory CD type 1 (RCD1) refers to those patients on gluten-free diet who have persistent enteropathy [14]. RCD1 patients often have negative serology for GRD a fact that distinguishes them from those patients with persistent enteropathy due to on-going exposure to gluten (dietary indiscretions). A subgroup of patients has evidence of an abnormal population of intraepithelial lymphocytes. This group is designated refractory CD type 2 (RCD2) [15]. Seven of our patients appear to belong to the RCD1 group, two to RCD2 both of which died of enteropathy-associated lymphoma. Patients with RCD2 have a higher risk of developing lymphoma (37%) by comparison to those with RCD1 (14%). It is unlikely that the neurological manifestations in our patient with biopsy proven metastatic enteropathy-associated lymphoma represent a paraneoplastic phenomenon, as the lymphoma was diagnosed 10 years after the neurological presentation.

RCD is rare as is cortical myoclonus with ataxia. This suggests that the two conditions are aetiologically linked rather than occurring by chance. The improvement of the ataxia following the introduction of gluten-free diet argues in favour of an aetiological link between the two. The new evidence for refractory CD, also explains the perception, from previous case reports, that this condition is unresponsive to gluten-free diet. This is in contrast to other neurological manifestations of gluten related disorders such as gluten ataxia (without myoclonus) that have been shown to be responsive to a strict gluten-free diet. (2) Whilst the ataxia in some of these patients did improve, it is the myoclonus that causes persistent disability.

**Figure 4:**
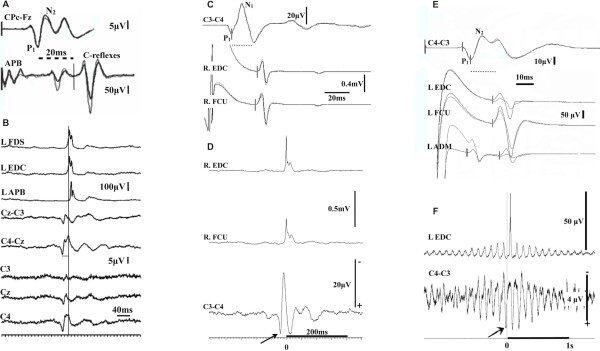
**Irregular spontaneous, action and reflex myoclonus/myoclonic tremor (cases 4, 6 and 8). (A)** ‘Giant’ somatosensory evoked potentials and C-reflex at a latency of 46 ms after stimulating median nerve at the wrist (case 4). The P1 precedes the C-reflex by 20 ms. **(B)** JLBA (245 sweeps) revealed a biphasic spike. There is co-contraction of agonist/antagonist and a proximodistal recruitment order. The EEG spike precedes the onset of the averaged EMG from the left APB by 20 ms – this is identical to the latency observed between the P1 waveform on the SEPs and the C-reflex from the APB **(C)** ‘Giant’ cortical waveforms and C-reflexes in the forearm muscles (latency of 42 ms) after stimulation of the median nerve at the wrist (case 6). The P1 component of the cortical waveform precedes the onset of the C-reflexes by 17 ms. **(D)** JLBA revealed a biphasic cortical correlate preceding the onset of the averaged and rectified EMG discharges (EDC). The latency from the positive EEG spike to the onset of the averaged EMG is very similar to the one recorded in the SEPs between the P1 component of the cortical waveform and the long loop responses in the forearm. **(E)** ‘Giant’ waveforms in the somatosensory evoked potentials (median nerve at the wrist) with prominent C-reflexes following by 16 ms (EDC) the P1 component of the cortical waveform (case 8). **(F)** JLBA from the left EDC while left arm at rest. The averaged data (531 sweeps) show a biphasic cortical correlate in the central region – the positive spike precedes the onset of the averaged EMG from the EDC by about 17 ms. ADM = abductor digiti minimi, APB = abductor pollicis brevis, EDC = extensor digitorum communis, FCU = flexor carpi ulnaris, FDS = Flexor digitorum superficialis, SEPs = somatosensory evoked potentials.

Clinically these patients differ from those with “myoclonic ataxia” (e.g. mitochondrial diseases, Baltic myoclonus, opsoclonus myoclonus ataxia syndrome, Creutzfeld-Jacob disease**,** post-hypoxia, toxic/metabolic myoclonus etc.) because their myoclonus is always focal (face or arm or leg) at onset but with time tends to spread to affect other parts of the body. Despite this, it tends to still remain asymmetric. This is in contrast to what is usually observed in the aforementioned disorders that can present with cortical myoclonus and ataxia where myoclonus tends to be more multifocal. In addition unlike gluten ataxia, the cerebellar ataxia tends to be less prominent with minimal atrophy on imaging. Three patients from this group presented at onset with focal, continuous and spontaneous myoclonus of cortical origin (e.g. Figure 2) fulfilling the definition of epilepsia partialis continua [11, 16, 17]. The focal motor jerks were initially restricted to one region but became more widespread with time and had features of cortical myoclonic tremor (Figure 4 and Additional file 1: Figure S1). The clinical variability seen in this entity included action, noise and stimulus reflex myoclonus affecting upper and lower limbs (Additional file 2: Figure S2 and Additional file 3: Figure S3), and episodes of Jacksonian march spreading from the areas most affected by myoclonic jerks, on occasions progressing to secondarily generalized tonic-clonic seizures.

Immunosuppression with mycophenolate appeared to help the cerebellar ataxia and in some patients improved the histological abnormalities on duodenal biopsy. The most disabling feature, however, remained the myoclonus. Anticonvulsants did, however prevent secondary generalised seizures in all patients who had experienced seizures at onset. Two of our patients who have recently started perampanel showed promising improvement of their myoclonus.

It appears that whatever immunological factors are associated with refractoriness of the enteropathy are also likely to be involved in the cortical hyperexcitability. Neuronal hyperexcitability may not be confined to the cortex as there is evidence that sensitivity to gluten may be implicated in another “hyperexcitable” neurological disease, that of stiff person syndrome [18]. The presence of TG6 antibodies in all 4 of the baseline samples available for testing does suggest that these antibodies may have a role to play in some of the neurological manifestation. However, the absence of transglutaminase antibodies (including TG6) and other gluten-related antibodies from the patients’ serum after strict gluten free diet, argues against these antibodies being directly involved in the generation of the myoclonus. This is despite the fact that transglutaminase antibodies (both TG2 and TG6) have been shown to induce ataxia in a mouse model [19].

Pathology from post-mortem material has confirmed that the cerebellum is commonly involved in such cases [4, 8, 20]. Additional information obtained from patients with gluten ataxia suggests that there is loss of Purkinje cells but also evidence of inflammation as indicated by perivascular cuffing with lymphocytes [8]. In one of our 9 patients where PM material was available, there was indeed evidence of Purkinje cell loss but no obvious inflammation. However this patient was already on immunosuppressive treatment. The role of the cerebellum in the generation of cortical myoclonus merits some consideration. It has been suggested that cortical hyperexcitability is due to enhanced facilitation of the cerebral motor cortex by the cerebellum [20]. Granule cells enhance activity of Purkinje cells whilst basket cells inhibit it. A greater relative loss of granule cells creates a mismatch between excitation and inhibition of Purkinje cells, with a prevalence of the latter. As a result, the cerebellar nuclei would be released from the inhibition of Purkinje cells resulting in facilitation of the cerebral motor cortex from the cerebellar nuclei. Whilst such a finding has been described in some cases of cortical myoclonus (two of which had CD) [20] in our single case where post mortem material was available we only found loss of Purkinje cells but intact granule and basket cells. Furthermore it is unclear why only very few patients with CD and ataxia will develop cortical hyperexcitability.

## Conclusion

These 9 cases illustrate the spectrum of cortical myoclonus with ataxia seen in refractory CD and reaffirm the concept that cortical myoclonus is a continuum, ranging from focal reflex jerks to spontaneous motor epilepsy [11, 21].

Based on our experience CD appears to be the commonest cause of cortical myoclonus. It is therefore important to consider CD as a potential diagnosis of all cases with cortical myoclonus manifesting as myoclonic tremor and/or epilepsia partialis continua. It is also essential to be aware of the refractoriness of the enteropathy and the need for immunosupression.

## Methods

All 9 patients were identified initially on clinical and then neurophysiological grounds from a cohort of over 600 patients regularly attending the gluten/neurology clinic based at the Royal Hallamshire Hospital, Sheffield UK. This is an observational study. All the diagnostic tests performed and treatments given were part of routine patient clinical care. The local ethics committee (Sheffield Teaching Hospitals) has cleared this in writing.

The Xltek EMU128 Headbox (Optima Medical Ltd) multichannel amplifier was used for EEG and surface EMG recordings. Sampling frequency of 2 Khz was used. EMG data was band pass filtered at 50-1000 Hz and EEG channels at 0.5 – 70 Hz. The stored data used for quantitative analysis, including JLBA did not undergo filtering, except that initially performed by the amplifier during data acquisition (0.15 – 940 Hz).

The technique of JLBA was applied based on previous reports [11, 12]. An offline analysis of patient data was performed by using the data available from the EEG and EMG polygraphic recordings, after exporting them in ‘EDF’ format. Spike 2 (version 7) software was used to analyse the data. EMG channels were rectified and DC removed (time constant 0.05) to allow identification of a clear onset of the EMG discharges. EEG data was not filtered. This enabled us to be able to record slow pre-movement cortical potentials that can be seen during voluntary movements. Events were marked from small duration (<50 ms) myoclonic jerks, captured from the surface EMG electrodes. Several hundred to several thousand events were collected for each patient (range 700–10,000). These events were then used for averaging, JLBA of the EEG and surface EMG polygraphic data. SEP recordings were made according to recent recommendations [13]. The presence of C-reflexes was assessed with additional surface electrodes in the forearm and leg muscles and a single muscle in the hand and foot.

## Electronic supplementary material

Additional file 1: Figure S1: Cortical myoclonic tremor (case 2). **(A)** EEG and polygraphic recordings with multiple surface EMG electrodes from the patient’s right upper limb. Continuous myoclonic jerks at a frequency of ≈ 6 Hz are synchronously affecting proximal and distal muscles. There is very fast proximodistal recruitment with simultaneous co-activation of pairs of antagonists. The duration of the EMG discharges is very short, on average less than 30 ms. **(B)** JLBA from the right BB. Two sets of independent averages were superimposed (844 and 648 sweeps were used). A sharp, spiky positive–negative EEG correlate appears in the contralateral central region, preceding the onset of the averaged EMG discharges by ≈ 18 ms. APB = abductor pollicis brevis, BB = biceps brachii, EDC = extensor digitorum communis, FDI = first dorsal interosseus, FDS = flexor digitorum superficialis, TB = triceps brachii. (TIFF 19 MB)

Additional file 2: Figure S2: Spontaneous and action myoclonus/myoclonic tremor (cases 3 and 5). **(A)** Somatosensory evoked potentials from the legs (case 3) produced grossly asymmetrical cortical waveforms (>50%). Prominent action myoclonus was seen on clinical examination, only from the right leg. However, note the absence of C-reflexes. **(B)** JLBA (3000 sweeps) from the right ADM (case 3) shows a biphasic positive–negative cortical correlate in the contralateral central region. There is phase reversal around C3 in the bipolar montages. The latency between the cortical positive spike at C3 and the onset of the EMG bursts from the ADM is ≈ 23 ms. **(C)** Electrical stimulation of the left median nerve at the wrist (case 5) showed normal amplitude cortical waveforms. There are some low amplitude long loop reflexes appearing in the forearm flexors and extensors and the abductor pollicis brevis at a latency of 50 and 55 ms, respectively. The patient is affected by a large fibre axonal peripheral neuropathy and is 1,91 m tall. Therefore, these latencies would be in keeping with low amplitude cortical reflexes. **(D)** A positive spike appears in the JLBA (4,309 sweeps) in case 5. The positive spike is maximal in the left frontocentral cortical electrodes, better formed at F3C3. The peak of the positive spikes precedes the onset of the averaged EMG discharges from the right APB by ≈ 16 ms, pointing towards a fast corticospinal transmission. Note the very low amplitude of the positive spikes (<1 μV). However, these are clearly standing out from the background due to the high number of averaged sweeps, resulting in very substantial increase in the signal-to-noise ratio. AH = abductor hallucis, APB = abductor pollicis brevis, FDI = first dorsal interosseus, EDC = extensor digitorum communis, GST = gastrocnemious, TA = tibialis anterior. (TIFF 5 MB)

Additional file 3: Figure S3: Lower limb action and reflex cortical myoclonus (case 9). **(A)** Somatosensory evoked potentials after electrical stimulation of the left posterior tibial nerve. The cortical waveform is ‘Giant’, above 20 μV in amplitude and there are conspicuous long loop reflexes affecting the lower leg flexor/extensors, with latency from the electrical stimulus at the ankle of 86 ms. **(B)** The Jerk-locked back averaging from the left TA (76 sweeps) shows biphasic, positive/negative EEG spikes, with the positive spikes lagging behind the onset of the averaged EMG from the TA by around 30 ms. TA = tibialis anterior, GST = gastrocnemious, AH = abductor hallucis. (TIFF 4 MB)
