# Supplementary figures and images for: Myoclonus ataxia and refractory coeliac disease
Source: Cerebellum Ataxias. 2014 Sep 1;1:11. doi: 10.1186/2053-8871-1-11 (PMC4552176; doi:10.1186/2053-8871-1-11)

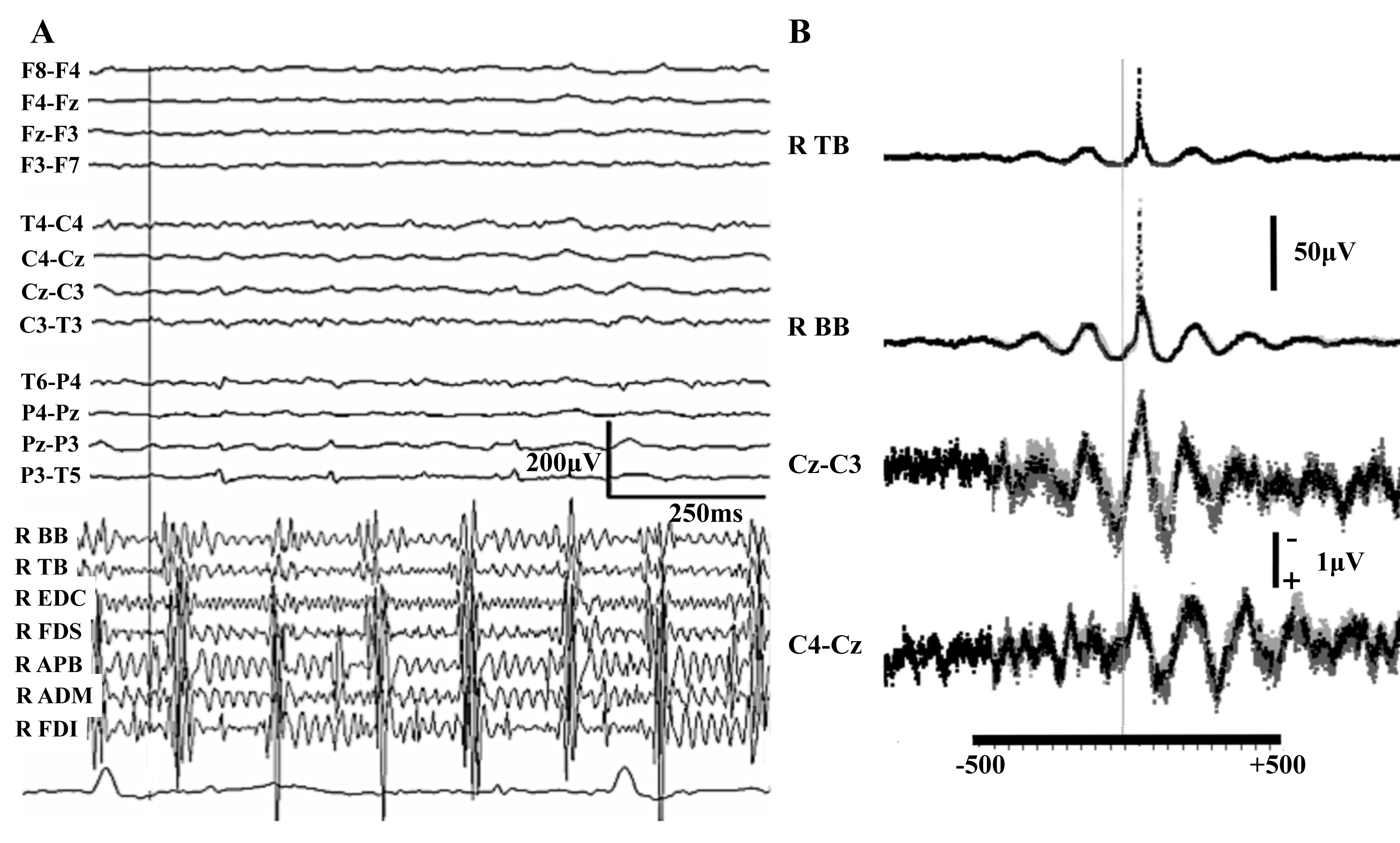

Supplement: Supplementary file 1 — Additional file 1: Figure S1: Cortical myoclonic tremor (case 2). (A) EEG and polygraphic recordings with multiple surface EMG electrodes from the patient’s right upper limb. Continuous myoclonic jerks at a frequency of ≈ 6 Hz are synchronously affecting proximal and distal muscles. There is very fast proximodistal recruitment with simultaneous co-activation of pairs of antagonists. The duration of the EMG discharges is very short, on average less than 30 ms. (B) JLBA from the right BB. Two sets of independent averages were superimposed (844 and 648 sweeps were used). A sharp, spiky positive–negative EEG correlate appears in the contralateral central region, preceding the onset of the averaged EMG discharges by ≈ 18 ms. APB = abductor pollicis brevis, BB = biceps brachii, EDC = extensor digitorum communis, FDI = first dorsal interosseus, FDS = flexor digitorum superficialis, TB = triceps brachii. (TIFF 19 MB) [file 40673_2014_12_MOESM1_ESM.tiff]

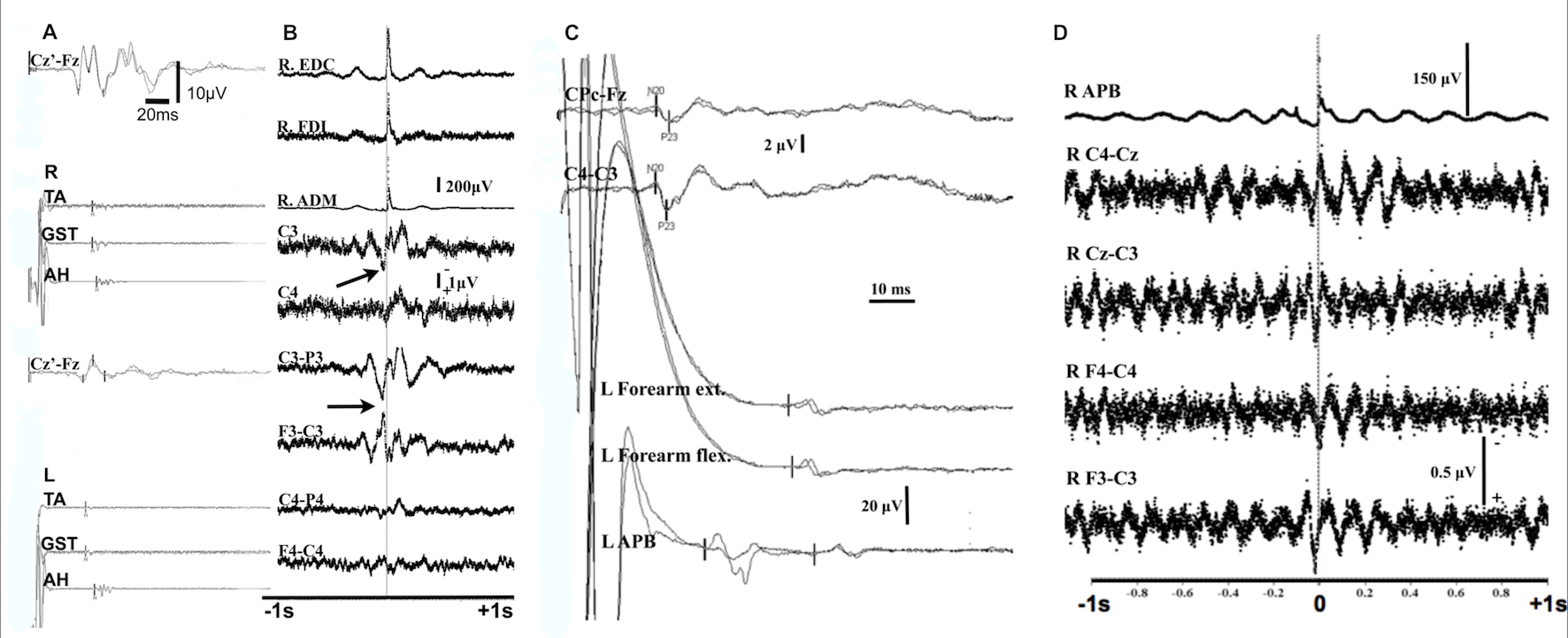

Supplement: Supplementary file 2 — Additional file 2: Figure S2: Spontaneous and action myoclonus/myoclonic tremor (cases 3 and 5). (A) Somatosensory evoked potentials from the legs (case 3) produced grossly asymmetrical cortical waveforms (>50%). Prominent action myoclonus was seen on clinical examination, only from the right leg. However, note the absence of C-reflexes. (B) JLBA (3000 sweeps) from the right ADM (case 3) shows a biphasic positive–negative cortical correlate in the contralateral central region. There is phase reversal around C3 in the bipolar montages. The latency between the cortical positive spike at C3 and the onset of the EMG bursts from the ADM is ≈ 23 ms. (C) Electrical stimulation of the left median nerve at the wrist (case 5) showed normal amplitude cortical waveforms. There are some low amplitude long loop reflexes appearing in the forearm flexors and extensors and the abductor pollicis brevis at a latency of 50 and 55 ms, respectively. The patient is affected by a large fibre axonal peripheral neuropathy and is 1,91 m tall. Therefore, these latencies would be in keeping with low amplitude cortical reflexes. (D) A positive spike appears in the JLBA (4,309 sweeps) in case 5. The positive spike is maximal in the left frontocentral cortical electrodes, better formed at F3C3. The peak of the positive spikes precedes the onset of the averaged EMG discharges from the right APB by ≈ 16 ms, pointing towards a fast corticospinal transmission. Note the very low amplitude of the positive spikes (<1 μV). However, these are clearly standing out from the background due to the high number of averaged sweeps, resulting in very substantial increase in the signal-to-noise ratio. AH = abductor hallucis, APB = abductor pollicis brevis, FDI = first dorsal interosseus, EDC = extensor digitorum communis, GST = gastrocnemious, TA = tibialis anterior. (TIFF 5 MB) [file 40673_2014_12_MOESM2_ESM.tiff]

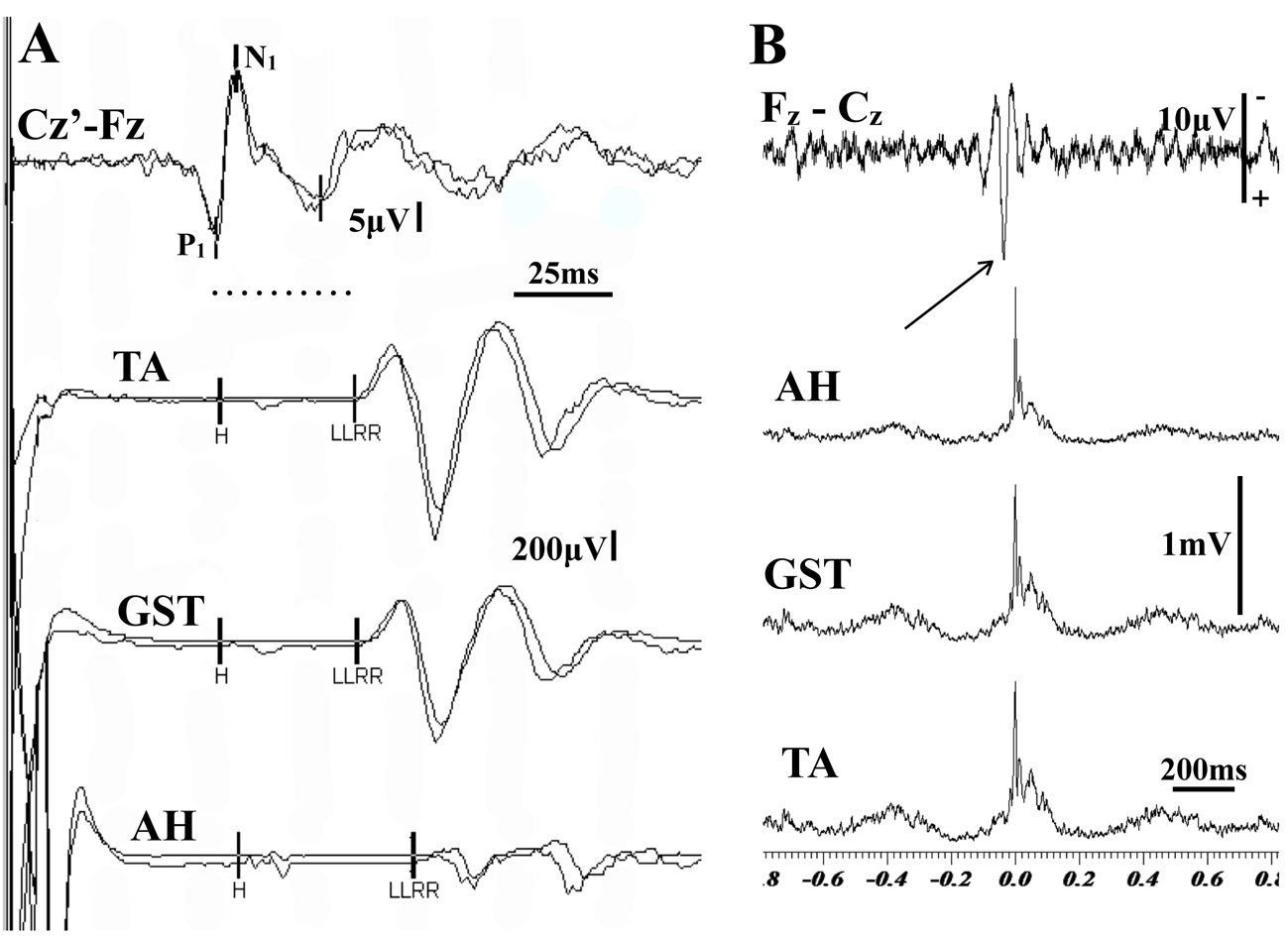

Supplement: Supplementary file 3 — Additional file 3: Figure S3: Lower limb action and reflex cortical myoclonus (case 9). (A) Somatosensory evoked potentials after electrical stimulation of the left posterior tibial nerve. The cortical waveform is ‘Giant’, above 20 μV in amplitude and there are conspicuous long loop reflexes affecting the lower leg flexor/extensors, with latency from the electrical stimulus at the ankle of 86 ms. (B) The Jerk-locked back averaging from the left TA (76 sweeps) shows biphasic, positive/negative EEG spikes, with the positive spikes lagging behind the onset of the averaged EMG from the TA by around 30 ms. TA = tibialis anterior, GST = gastrocnemious, AH = abductor hallucis. (TIFF 4 MB) [file 40673_2014_12_MOESM3_ESM.tiff]
